# The CFII components PCF11 and Cbc change subnuclear localization as cells differentiate in an adult stem cell lineage

**DOI:** 10.17912/micropub.biology.001718

**Published:** 2025-08-15

**Authors:** Iliana Nava, Margaret T. Fuller, Lorenzo Gallicchio

**Affiliations:** 1 Department of Developmental Biology, Stanford University, Stanford, California, United States; 2 Department of Genetics, Stanford University, Stanford, California, United States

## Abstract

Stage specific increased expression of PCF11 and Cbc, the two components of Cleavage Factor complex II, contribute to developmentally regulated 3’UTR shortening due to alternate polyadenylation of nascent RNA molecules in *Drosophila* spermatocytes. Here, we show that both Cbc and PCF11 change subnuclear localization during male germ cell differentiation, from homogeneous in the nucleus in spermatogonia and early spermatocytes to concentrated around the nucleolus in later spermatocyte stages.

**Figure 1. PCF11 and Cbc change localization from homogeneous in the nucleoplasm to more concentrated at the nucleolar periphery as spermatocytes grow:**
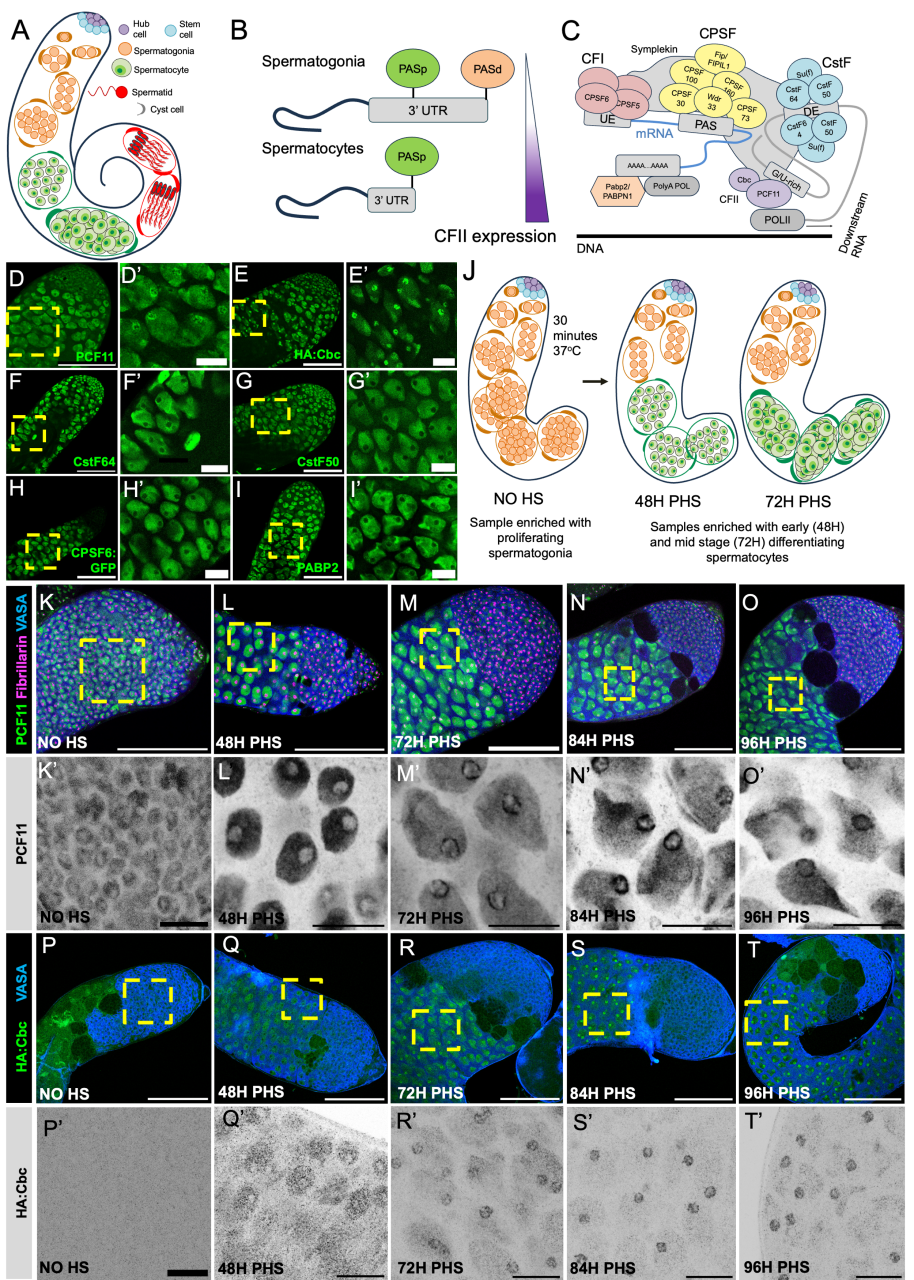
(A) Schematic of a *Drosophila* testis. (B) Diagram of the APA switch observed as spermatogonia differentiate into spermatocytes. PASd (orange): the distal PAS processed in spermatogonia. PASp (green): the proximal PAS processed in spermatocytes. The APA switch is at least partially regulated by increased expression of CFII in spermatocytes compared to spermatogonia. (C) Diagram of the cleavage machinery and its complexes: CPSF (yellow), CstF in (blue), CFI (red) and CFII in (purple). UE = Upstream Element, DE = Downstream Element. (D-I) Apical tips of fixed *Drosophila* *melanogaster* testes immuno-stained for: (D-D’) PCF11, (E-E’) HA:Cbc, (F-F’) CstF64, (G-G’) CstF50, (H-H’) CPSF6:GFP, (I-I’) PABP2. Scalebars are 100 uM (D-I) and 20 uM (D’-I’). Genotypes: *w1118* (D-F-G-I), *HA:Cbc* (E), *CPSF6:GFP* (VDRC 318105) (J) Diagram of the heat shock time course system for synchronous spermatocyte differentiation. PHS = post heat shock. (K-O) Apical tips of fixed *Drosophila* *melanogaster* testes immuno-stained with antibodies against: PCF11 (green), Fibrillarin (magenta) and VASA (blue). Timepoints: (K) no heat shock, (L) 48H PHS, (M) 72H PHS, (N) 84H PHS, (O) 96H PHS. Scalebars are: 100 uM (K-O) and 20 uM (K’-O’). (P-T) Apical tips of fixed *Drosophila melanogaster* testes immuno-stained with antibodies against: HA:Cbc (green), and VASA (blue). Timepoints: (P) no heat shock, (Q) 48H PHS, (R) 72H PHS, (S) 84H PHS, (T) 96H PHS. Scalebars are: 100 uM (P-T) and 20 uM (P’-T’).

## Description

Co-transcriptional Cleavage and Polyadenylation of nascent RNA molecules is the final step of transcription and is required for the newly transcribed mRNAs to be exported to the cytoplasm (Rodríguez-Molina et al., 2023). A multi subunit cleavage machinery complex responsible for making the 3’end cut directs cleavage and polyadenylation to specific sites on the nascent RNA by recognizing specific hexametric RNA motifs termed Polyadenylation Signals (PAS). The canonical PAS (AAUAAA) and some variants considered “strong” are more frequently processed by the cleavage machinery than “weak” variants or non-canonical PAS (Gallicchio et al., 2023; Gruber & Zavolan, 2019; Neve et al., 2017). Genes in multicellular eukaryotes usually possess multiple PAS motifs, and the same genetic locus often gives rise to alternative mRNA isoforms that differ at their 3’ end due to a different choice of which PAS is utilized, a process named Alternative Polyadenylation (APA). An alternate PAS located upstream of a stop codon can result in a truncated or variant protein. Most commonly, alternate PAS sites located in the 3’ untranslated region (3’UTR) can result in mRNA isoforms that differ in 3’UTR length and content. As 3’UTRs are a hub of *cis* -regulatory sequences that attract *trans* -acting factors such as microRNAs and RNA binding proteins, changes in the 3’UTR can affect mRNA stability, localization and translation (Gallicchio et al., 2023). Thus, APA can profoundly affect what proteins are expressed by simply changing the site at which transcripts are terminated.

Changes in APA have been implied in numerous disease states (Davis et al., 2022; Gruber & Zavolan, 2019; Kurozumi & Lupold, 2021; Ren et al., 2020; Tian & Manley, 2016; Venkat et al., 2020; Y. Zhang et al., 2021). For example, certain cancer cell lines tend to express mRNAs with shorter 3’UTRs, causing loss of repressors (i.e. miRNAs) binding sites and subsequent abnormal upregulation of the encoded proteins (Mayr & Bartel, 2009; Y. Zhang et al., 2021). Recently, APA has been shown to be a feature of many healthy biological processes, and that APA must be tightly regulated to maintain cell and tissue homeostasis (Gallicchio et al., 2023; Kasowitz et al., 2018; Smibert et al., 2012; Ulitsky et al., 2012; Vallejos Baier et al., 2017; Yang et al., 2022).

Developmentally regulated processing of alternate PAS sites can program cell type specific changes in 3’UTRs that profoundly alter where and when certain proteins are expressed. For example, differentiating neurons tend to express mRNA isoforms with longer 3’UTRs due to APA (Oktaba et al., 2015; Vallejos Baier et al., 2017). This may be particularly important for proper sub-cellular localization of specific mRNAs in neurons (Arora et al., 2022). Conversely, differentiating spermatocytes tend to express mRNA isoforms with shorter 3’UTRs compared to the transcripts expressed from the same genes in spermatogonia (Berry et al., 2022; Li et al., 2016; Shan et al., 2017). In *Drosophila* spermatocytes, more than 500 genes switch from a distal cleavage site (used in spermatogonia) to a more proximal one due to developmentally regulated APA (Berry et al., 2022) (Fig 1 A-B). Strikingly, the resulting switch in 3’UTR length correlated with changes in protein expression: in some cases, the protein was not detected in spermatogonia but highly translated in spermatocytes, while in others the encoded protein was translated in spermatogonia but not detected in spermatocytes (Berry et al., 2022).

The tendency to cleave nascent transcripts at more proximal PASs in *Drosophila* spermatocytes was in part due to cell type specific upregulation of specific components of the cleavage machinery. The cleavage machinery is composed of 4 major protein complexes: CPSF (Cleavage and Polyadenylation Specificity Factor), CstF (Cleavage Stimulation Factor), CFI (Cleavage factor I) and CFII (Cleavage Factor II), plus the non-complex proteins Symplekin, PolyA Polymerase and PABP2 (Gallicchio et al., 2023). Genetic experiments showed that high levels of the two components of CFII, PCF11 and Cbc, were required in spermatocytes to direct cleavage at a proximal site rather than the PAS used in spermatogonia for over 200 genes (Gallicchio et al., 2024). Cbc had been recently reported to change sub-nuclear localization as spermatocytes differentiate in *Drosophila* (Wu et al., 2021). While in spermatogonia Cbc was homogenously distributed in the nucleus, in late spermatocytes Cbc protein concentrated particularly in and around the nucleolus (Wu et al., 2021) ( Fig. 1 D).

Here we show that PCF11 also changes sub-nuclear localization as germ cells differentiate ( Fig. 1 E). We did not observe such re-colocalization to the nucleolus for several other cleavage factors tested: CstF50, CstF64, PABP2 and CPSF6 ( Fig. 1 F-G-H-I). Analysis of *Drosophila* testes enriched for specific germ cell stages through an *in vivo* synchronous differentiation strategy identified the stage of spermatocyte differentiation at which the switch of sub-nuclear localization of Cbc and PCF11 occurred. During spermatogenesis, proliferating spermatogonia undergo four rounds of transient amplifying division, producing a clone of interconnected germ cells enclosed in an envelope of two squamous epithelial somatic support cells, forming a cyst ( Fig. 1 A). The 16 cells then enter premeiotic S phase and initiate the spermatocyte program of cell growth and transcriptional activation. The switch from spermatogonial proliferation to spermatocyte differentiation relies on function of the *bag of marbles* ( *bam* ) gene, with Bam protein required to reach a specific threshold to trigger the switch (Insco et al., 2009). Flies that completely lack *bam* have testes filled with cysts containing spermatogonia that undergo several extra rounds of mitotic division then die (Harris et al., 2025; Insco et al., 2009). Providing a brief pulse of Bam expression under control of a heat shock inducible promoter can induce *bam ^-/-^* spermatogonia to differentiate into spermatocytes, then eventually spermatids and sperm (Jongmin Kim et al., 2017). Collecting samples at different time points after heat shock yields testes containing semi-synchronous cohorts of spermatocyte cysts at specific stages of maturation, depending on the time after heat shock ( Fig. 1 J).

Immunofluorescence staining at different time points after heat shock revealed that as spermatocytes mature, PCF11 and Cbc, the two components of CFII, change sub-nuclear localization. In spermatogonia (no heat shock sample, Fig. 1 K-K’) PCF11 is homogenously distributed in the nucleoplasm. At 48 hours post heat shock (48H PHS) testes had newly formed *bam ^-/-^* spermatogonia at the apical tip and the rest of the testes were filled with early, polar spermatocytes. At this stage, PCF11 and Cbc were upregulated in spermatocytes compared to the juxtaposed spermatogonia but both proteins were homogeneously distributed in the spermatocyte nuclei and excluded from the nucleolus, similarly to spermatogonia ( Fig. 1 L and Q). In testes fixed at 72 H PHS, both PCF11 and Cbc showed higher immunofluorescence signal around the nucleolar periphery than in the general nucleoplasm (Fig. M and R). At later time points (84H and 96H PHS) the signal around the nucleolus intensified ( Fig. 1 N, S, and O, T, respectively) (see SOM 1-4 for additional examples).

The nucleolus, a key membrane-less nuclear compartment, is the site of processing of ribosomal RNA (rRNA) and ribosome assembly. Recently, transcription of rDNA spacer regions by PolII has been shown to have an active role in rRNA production and nucleolar architecture (Abraham et al., 2020; Caudron-Herger et al., 2016; Khosraviani et al., 2024). Consistent with active PolII dependent transcription near the nucleolus, the tTAFs (testis specific TATA binding protein Associated Factors), required for robust transcription at spermatocytes specific promoters, have been shown to concentrate at the nucleolus in spermatocytes (Chen et al., 2005). As differentiating spermatocytes grow 25 times in volume and also need to assemble many ribosomes to endow sufficient capability to translate the many new mRNAs necessary for the post meiotic morphological transformation to mature sperm, it is likely that transcription of rRNAs and their PolII dependent spacer regions is strongly upregulated in spermatocytes. PCF11 has been shown to bind to the C terminal domain of PolII and to regulate transcription termination (Kamieniarz-Gdula et al., 2019; Sadowski et al., 2003; Z. Zhang & Gilmour, 2006). We propose that the concentration of PCF11 and Cbc around the perimeter of the nucleolus in growing spermatocytes may be due to binding of PCF11 to active PolII, bringing Cbc with it. Whether PCF11 and/or Cbc have an active role in regulating rRNAs processing or transcription termination of rDNA spacer regions remains to be elucidated.

## Methods

*Drosophila lines and husbandry*

Flies were raised at either room temperature or 25 ^o^ C except as described for the heat shock (HS) time course. For HS time course experiments, *bam* ^1/86^ mutants carrying a *bam* transgene under the control of a heat shock promoter were heat-shocked at 37 ^o^ C for 30 minutes in late larval and pupal stages, then returned to 25 ^o^ C. Flies were collected at different time points after heat shock, and their testes were then dissected and fixed for immunofluorescence.

*Immunofluorescence and Imaging*

Testes were dissected from 0-3 day old male flies in 1X PBS at room temperature in a cyclops dissecting dish for a maximum of 10 minutes, transferred to an Eppendorf tube and immediately fixed in 5% formaldehyde in 1X PBS for 30 minutes with rocking at room temperature. Testes were then rinsed twice with 1X PBS and permeabilized by rocking at room temperature for 30 minutes in 0.3% Triton X-100 and 0.6% sodium deoxycholate in 1× PBS. The testes were then washed 3 times 15 minutes each in PBST (1× PBS + 0.05% Tween-20) with rocking at room temperature then blocked in 1X Western Blocking Reagent (WBR, Roche 11921673001) in PBST for 30 minutes rocking at room temperature. After blocking testes were incubated in primary antibody in 1X WBR in PBST overnight at 4 ^o^ C. The following day, testes were washed 3 times in PBST for 15 minutes each, rocking at room temperature, blocked again in 1X WBR in PBST for 30 minutes rocking at room temperature, and incubated for 2 hours with secondary antibody mix in 1X WBR in PBST with rocking at room temperature. Testes were then washed 3 times 15 minutes each in PBST, rocking at room temperature, then mounted on glass slides in 10 uL of ProLong Diamond antifade with DAPI (Thermo Fisher P36962). Slides were imaged on a Leica SP8 (Cell Science Imaging Facility (CSIF), Stanford University) and Images were processed with FIJI. The localization pattern for PCF11 and Cbc was observed in the following number of samples (indicated with n_p for PCF11 and n_c for Cbc): WT: n_p = 5, n_c = 5; no HS: n_p=9, n_c=3; 48H PHS: n_p=12, n_c=4; 72H PHS: n_p=14, n_c=4; 84H PHS: n_p=7, n_c=3; 96H PHS: n_p=10, n_c=3. The reported localization was observed in every replicate.

## Reagents

Flies used in this study were: *w1118* (Bloomington *Drosophila* Stock Center [BL] 3605), *CPSF6:GFP* (Vienna *Drosophila* Resource Center [VDRC] 318105), *HA:cbc* (kindly gifted from the Wang lab (Wu et al., 2021), *hsBam* / *CyO* ; *bam ^86^* , *bamGal4/TM6B(Jongmin Kim et al., 2017; D. McKearin & Ohlstein, 1995)* and *bam ^1^* / *TM6B (D. M. McKearin & Spradling, 1990)* .

Antibodies used: rabbit anti-CstF64 (1:400; a kind gift from Zbigniew Dominski, (Skrajna et al., 2018)); guinea pig anti-CstF50 (1:300; a kind gift from John T. Lis, (Ni et al., 2008)); chicken anti-GFP (1:10,000; Abcam 13970); rabbit anti-PABP2 (1:500; a kind gift from Martine Simonelig (Benoit et al., 1999)); rabbit anti-PCF11 (1:200; a kind gift from David Scott Gilmour, (Z. Zhang & Gilmour, 2006)); rabbit anti-HA (1:500; Cell Signaling Technologies 3724), mouse anti-HA (1:200; Biolegends 901501); mouse anti-Fibrillarin (1:300, Abcam ab4566); goat anti-Vasa (1:200; Santa Cruz Biotechnology sc-26877, discontinued); rat anti-Vasa (1:10 for supernatant or 1:100 for the concentrated version; both from Developmental Studies Hybridoma Bank antibody ID 760351). Secondary antibodies used were: donkey or goat anti-rabbit; anti-mouse; anti-guinea pig; anti-chicken; anti-rat; and anti-goat; all used at 1:500 and conjugated with either Alexa 488, Alexa 568, Alexa 594, or Alexa 647 (Thermo Fisher catalog).

## References

[R1] Abraham Karan J., Khosraviani Negin, Chan Janet N. Y., Gorthi Aparna, Samman Anas, Zhao Dorothy Y., Wang Miling, Bokros Michael, Vidya Elva, Ostrowski Lauren A., Oshidari Roxanne, Pietrobon Violena, Patel Parasvi S., Algouneh Arash, Singhania Rajat, Liu Yupeng, Yerlici V. Talya, De Carvalho Daniel D., Ohh Michael, Dickson Brendan C., Hakem Razq, Greenblatt Jack F., Lee Stephen, Bishop Alexander J. R., Mekhail Karim (2020). Nucleolar RNA polymerase II drives ribosome biogenesis. Nature.

[R2] Arora, A., Goering, R., Lo, H. Y. G., Lo, J., Moffatt, C., & Taliaferro, J. M. (2022). The Role of Alternative Polyadenylation in the Regulation of Subcellular RNA Localization. *Frontiers in Genetics* , *Volume 12-2021* . https://www.frontiersin.org/journals/genetics/articles/10.3389/fgene.2021.818668 10.3389/fgene.2021.818668 PMC8795681 35096024

[R3] Benoit B (1999). The Drosophila poly(A)-binding protein II is ubiquitous throughout Drosophila development and has the same function in mRNA polyadenylation as its bovine homolog in vitro. Nucleic Acids Research.

[R4] Berry Cameron W., Olivares Gonzalo H., Gallicchio Lorenzo, Ramaswami Gokul, Glavic Alvaro, Olguín Patricio, Li Jin Billy, Fuller Margaret T. (2022). Developmentally regulated alternate 3′ end cleavage of nascent transcripts controls dynamic changes in protein expression in an adult stem cell lineage. Genes & Development.

[R5] Caudron-Herger Maïwen, Pankert Teresa, Rippe Karsten (2016). Regulation of nucleolus assembly by non-coding RNA polymerase II transcripts. Nucleus.

[R6] Chen Xin, Hiller Mark, Sancak Yasemin, Fuller Margaret T. (2005). Tissue-Specific TAFs Counteract Polycomb to Turn on Terminal Differentiation. Science.

[R7] Davis Amanda G., Johnson Daniel T., Zheng Dinghai, Wang Ruijia, Jayne Nathan D., Liu Mengdan, Shin Jihae, Wang Luyang, Stoner Samuel A., Zhou Jie-Hua, Ball Edward D., Tian Bin, Zhang Dong-Er (2022). Alternative polyadenylation dysregulation contributes to the differentiation block of acute myeloid leukemia. Blood.

[R8] Gallicchio Lorenzo, Matias Neuza R., Morales-Polanco Fábian, Nava Iliana, Stern Sarah, Zeng Yi, Fuller Margaret T. (2024). A developmental mechanism to regulate alternative polyadenylation in an adult stem cell lineage. Genes & Development.

[R9] Gallicchio Lorenzo, Olivares Gonzalo H., Berry Cameron W., Fuller Margaret T. (2023). Regulation and function of alternative polyadenylation in development and differentiation. RNA Biology.

[R10] Gruber Andreas J., Zavolan Mihaela (2019). Alternative cleavage and polyadenylation in health and disease. Nature Reviews Genetics.

[R11] Harris Devon E., Kim Jongmin J., Stern Sarah R., Vicars Hannah M., Matias Neuza R., Gallicchio Lorenzo, Baker Catherine C., Fuller Margaret T. (2025). An RNA-binding regulatory cascade controls the switch from proliferation to differentiation in the
*Drosophila*
male germ cell lineage. Proceedings of the National Academy of Sciences.

[R12] Insco Megan L., Leon Arlene, Tam Cheuk Ho, McKearin Dennis M., Fuller Margaret T. (2009). Accumulation of a differentiation regulator specifies transit amplifying division number in an adult stem cell lineage. Proceedings of the National Academy of Sciences.

[R13] Jongmin Kim, Chenggang Lu, Shrividhya Srinivasan, Stephan Awe, Alexander Brehm, & Margaret T. Fuller. (2017). Blocking promiscuous activation at cryptic promoters directs cell type – specific gene expression. *Science* . 10.1126/science.aal3096 PMC5572561 28522526

[R14] Kamieniarz-Gdula Kinga, Gdula Michal R., Panser Karin, Nojima Takayuki, Monks Joan, Wiśniewski Jacek R., Riepsaame Joey, Brockdorff Neil, Pauli Andrea, Proudfoot Nick J. (2019). Selective Roles of Vertebrate PCF11 in Premature and Full-Length Transcript Termination. Molecular Cell.

[R15] Kasowitz Seth D., Ma Jun, Anderson Stephen J., Leu N. Adrian, Xu Yang, Gregory Brian D., Schultz Richard M., Wang P. Jeremy (2018). Nuclear m6A reader YTHDC1 regulates alternative polyadenylation and splicing during mouse oocyte development. PLOS Genetics.

[R16] Khosraviani Negin, Yerlici V. Talya, St-Germain Jonathan, Hou Yi Yang, Cao Shi Bo, Ghali Carla, Bokros Michael, Krishnan Rehna, Hakem Razqallah, Lee Stephen, Raught Brian, Mekhail Karim (2024). Nucleolar Pol II interactome reveals TBPL1, PAF1, and Pol I at intergenic rDNA drive rRNA biogenesis. Nature Communications.

[R17] Kurozumi Akira, Lupold Shawn E. (2021). Alternative polyadenylation: An untapped source for prostate cancer biomarkers and therapeutic targets?. Asian Journal of Urology.

[R18] Li Wencheng, Park Ji Yeon, Zheng Dinghai, Hoque Mainul, Yehia Ghassan, Tian Bin (2016). Alternative cleavage and polyadenylation in spermatogenesis connects chromatin regulation with post-transcriptional control. BMC Biology.

[R19] Mayr Christine, Bartel David P. (2009). Widespread Shortening of 3′UTRs by Alternative Cleavage and Polyadenylation Activates Oncogenes in Cancer Cells. Cell.

[R20] McKearin D M, Spradling A C (1990). bag-of-marbles: a Drosophila gene required to initiate both male and female gametogenesis.. Genes & Development.

[R21] Neve Jonathan, Patel Radhika, Wang Zhiqiao, Louey Alastair, Furger André Martin (2017). Cleavage and polyadenylation: Ending the message expands gene regulation. RNA Biology.

[R22] Ni Zhuoyu, Saunders Abbie, Fuda Nicholas J., Yao Jie, Suarez Jose-Ramon, Webb Watt W., Lis John T. (2008). P-TEFb Is Critical for the Maturation of RNA Polymerase II into Productive Elongation In Vivo. Molecular and Cellular Biology.

[R23] Oktaba Katarzyna, Zhang Wei, Lotz Thea Sabrina, Jun David Jayhyun, Lemke Sandra Beatrice, Ng Samuel Pak, Esposito Emilia, Levine Michael, Hilgers Valérie (2015). ELAV Links Paused Pol II to Alternative Polyadenylation in the Drosophila Nervous System. Molecular Cell.

[R24] Ren Fanggang, Zhang Na, Zhang Lan, Miller Eric, Pu Jeffrey J. (2020). Alternative Polyadenylation: a new frontier in post transcriptional regulation. Biomarker Research.

[R25] Rodríguez-Molina Juan B., West Steven, Passmore Lori A. (2023). Knowing when to stop: Transcription termination on protein-coding genes by eukaryotic RNAPII. Molecular Cell.

[R26] Sadowski M. (2003). Independent functions of yeast Pcf11p in pre-mRNA 3' end processing and in transcription termination. The EMBO Journal.

[R27] Shan Lingjuan, Wu Chan, Chen Di, Hou Lei, Li Xin, Wang Lixia, Chu Xiao, Hou Yifeng, Wang Zhaohui (2017). Regulators of alternative polyadenylation operate at the transition from mitosis to meiosis. Journal of Genetics and Genomics.

[R28] Skrajna Aleksandra, Yang Xiao-cui, Dadlez Michał, Marzluff William F, Dominski Zbigniew (2018). Protein composition of catalytically active U7-dependent processing complexes assembled on histone pre-mRNA containing biotin and a photo-cleavable linker. Nucleic Acids Research.

[R29] Smibert Peter, Miura Pedro, Westholm Jakub O., Shenker Sol, May Gemma, Duff Michael O., Zhang Dayu, Eads Brian D., Carlson Joe, Brown James B., Eisman Robert C., Andrews Justen, Kaufman Thomas, Cherbas Peter, Celniker Susan E., Graveley Brenton R., Lai Eric C. (2012). Global Patterns of Tissue-Specific Alternative Polyadenylation in Drosophila. Cell Reports.

[R30] Tian Bin, Manley James L. (2016). Alternative polyadenylation of mRNA precursors. Nature Reviews Molecular Cell Biology.

[R31] Ulitsky Igor, Shkumatava Alena, Jan Calvin H., Subtelny Alexander O., Koppstein David, Bell George W., Sive Hazel, Bartel David P. (2012). Extensive alternative polyadenylation during zebrafish development. Genome Research.

[R32] Vallejos Baier Raul, Picao-Osorio Joao, Alonso Claudio R. (2017). Molecular Regulation of Alternative Polyadenylation (APA) within the Drosophila Nervous System. Journal of Molecular Biology.

[R33] Venkat Swati, Tisdale Arwen A., Schwarz Johann R., Alahmari Abdulrahman A., Maurer H. Carlo, Olive Kenneth P., Eng Kevin H., Feigin Michael E. (2020). Alternative polyadenylation drives oncogenic gene expression in pancreatic ductal adenocarcinoma. Genome Research.

[R34] Wu Jianbo, Li Xin, Gao Zhiyang, Pang Lin, Liu Xian, Huang Xiahe, Wang Yingchun, Wang Zhaohui (2021). RNA kinase CLP1/Cbc regulates meiosis initiation in spermatogenesis. Human Molecular Genetics.

[R35] Yang Yanbo, Wu Xiaohong, Yang Wenqian, Jin Weiwei, Wang Dongyang, Yang Jianye, Jiang Guanghui, Zhang Wen, Niu Xiaohui, Gong Jing (2022). Dynamic alternative polyadenylation during iPSC differentiation into cardiomyocytes. Computational and Structural Biotechnology Journal.

[R36] Zhang Yi, Liu Lian, Qiu Qiongzi, Zhou Qing, Ding Jinwang, Lu Yan, Liu Pengyuan (2021). Alternative polyadenylation: methods, mechanism, function, and role in cancer. Journal of Experimental & Clinical Cancer Research.

[R37] Zhang Zhiqiang, Gilmour David S. (2006). Pcf11 Is a Termination Factor in Drosophila that Dismantles the Elongation Complex by Bridging the CTD of RNA Polymerase II to the Nascent Transcript. Molecular Cell.

